# Fusion of a Primary Maxillary Central Incisor With a Mesiodens: A Case Report

**DOI:** 10.7759/cureus.108401

**Published:** 2026-05-06

**Authors:** Evangelos Vardakis, Efstathia Afrati

**Affiliations:** 1 Department of Paediatric Dentistry, National and Kapodistrian University of Athens, Athens, GRC; 2 Department of Prosthodontics, National and Kapodistrian University of Athens, Athens, GRC

**Keywords:** dental fusion, developmental anomalies, mesiodens, pediatric dentistry, primary dentition, supernumerary tooth, tooth extraction

## Abstract

Dental fusion is a developmental anomaly that involves the joining of two developing tooth germs. Although fusion between adjacent teeth has been widely reported, the fusion between a primary tooth and a supernumerary in the primary dentition remains uncommon. It has been reported in isolated case reports without relevant epidemiological studies. We present the case of a 3.5-year-old boy with pain in the upper anterior region. Clinical and radiographic examination revealed fusion between the primary maxillary right central incisor (51) and a mesiodens. Due to symptoms and the extent of carious lesions of the fused tooth, extraction was performed. This case highlights the diagnostic challenges in identifying this anomaly, the differentiation from gemination, and the importance of long-term monitoring of the erupting permanent successor.

## Introduction

Dental fusion is a developmental anomaly that occurs due to the fusion of two separately growing tooth germs during odontogenesis. In the different stages of odontogenesis, fusion can be complete, which involves the whole crown and root, or incomplete, involving only a part of the crown or the root [1,2]. Fusion affects 0.5%-2.5% of the primary dentition, a higher rate than the 0.1% observed in permanent teeth [3,4].

Fusion can occur between two adjacent teeth or between a normal tooth and a supernumerary tooth [1]. A mesiodens, the most common type of supernumerary tooth, is located in the premaxillary midline between the two central incisors, with a prevalence of 0.15%-1.9% in the pediatric population [5]. Although these anomalies are independent, their co-occurrence, the fusion of a primary tooth with a mesiodens, specifically remains uncommon as a distinct clinical entity, with minimal population-level prevalence data available for this particular combination [3]. In cases where supernumerary teeth are present, a syndromic or genetic background should be considered. Conditions associated with supernumerary teeth include cleidocranial dysplasia, Gardner syndrome, and Robinow syndrome [6].

The etiology of fusion remains unclear, though the most accepted theory is that physical forces or pressure cause contact between developing tooth germs during the proliferation stage of odontogenesis, leading to union of the enamel, dentine, and, in some cases, the pulp [1,3]. Clinically, fused teeth have numerous challenges such as aesthetic concerns, increased caries incidence along the fusion groove, space discrepancies, and potential effects on permanent successors [2,7]. The differentiation between fusion and gemination can be difficult. In fusion, counting the teeth reveals one missing tooth when the anomalous tooth is counted as one. On the contrary, in gemination, the number of teeth remains the same [8].

We present a case of fusion between the primary right central incisor (51) and a mesiodens in a 3.5-year-old boy. This report aims to describe the clinical and radiographic features of this anomaly, discuss its differentiation from gemination, outline the management rationale, and highlight the clinical significance of this presentation for the developing permanent dentition, including the risk of permanent tooth agenesis and the implications for long-term monitoring. This case is particularly relevant for general dental practitioners and pediatric dentists who may encounter anomalous anterior primary teeth and need to navigate the diagnostic and treatment decision-making process in young, often uncooperative patients.

## Case presentation

A 3.5-year-old boy presented to our clinic with a complaint of pain in the upper front teeth for two weeks due to an anomalous tooth, as mentioned by his parents. The patient’s medical history was non-contributory. Family history did not reveal dental abnormalities, and no features of an underlying syndrome were identified clinically, suggesting an isolated developmental anomaly. There was also no history of dental or facial trauma. Written informed consent was obtained from the patient’s parents for treatment, clinical photography and publication of this case report and its accompanying images.

Clinical examination

Extraoral examination did not reveal anything remarkable. Intraoral examination revealed a primary dentition consistent with the patient’s age. The right central incisor appeared wider mesiodistally than normal, with a visible groove on both the labial and palatal surfaces of the crown (Figure 1). The tooth was discolored, with deep carious lesions (International Caries Detection and Assessment System (ICDAS) score 5-6) extending along the fusion groove on both the labial and palatal surfaces, clinically suggestive of pulpal involvement. The depth of the caries-free groove was measured at 1 mm. The pain was reported by the parents as spontaneous and intermittent, worsening upon cold stimuli. Pulp vitality testing was not performed given the patient’s age and limited cooperation. Clinical signs such as spontaneous pain and deep carious lesions were considered sufficient to establish a diagnosis of pulpal pathology. The mesiodistal width of the fused tooth was measured clinically at approximately 10 mm, markedly wider than that of the contralateral central incisor (61), which measured approximately 6.5 mm. The shape and size of tooth 61 appeared normal, and no other supernumerary teeth were observed clinically. Counting the anomalous tooth as one revealed a normal number of teeth in the maxillary arch, leading to a diagnosis of gemination or fusion with a supernumerary tooth.

**Figure 1:**
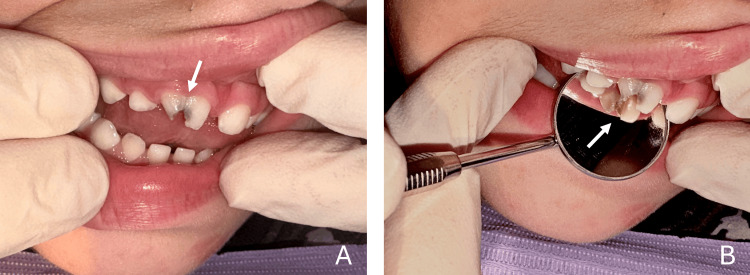
Intraoral clinical photograph (frontal view, maxillary anterior region). (A) Labial surface. (B) Palatal surface. The fused tooth (primary right central incisor 51 fused with mesiodens) is visible in the midline, with a clearly widened mesiodistal crown and a deep fusion groove (indicated by the arrows). The contralateral central incisor (61) appears normal in morphology.

Radiographic examination

An occlusal radiograph of the maxillary anterior region revealed an abnormal tooth structure, two root canals, and two completely fused roots (Figure 2). With these radiographic findings, we confirmed the fusion of the central incisor (51) with a supernumerary tooth. The permanent successor (tooth 11) was identified on the occlusal radiograph in its expected anatomical position, with no radiographic evidence of displacement or follicular enlargement. Its relationship to the apices of the fused primary tooth appeared undisturbed. Given the patient’s age and the principle of radiation dose in pediatric patients (as low as reasonably achievable, ALARA), advanced imaging (cone-beam CT, CBCT)) was not performed.

**Figure 2:**
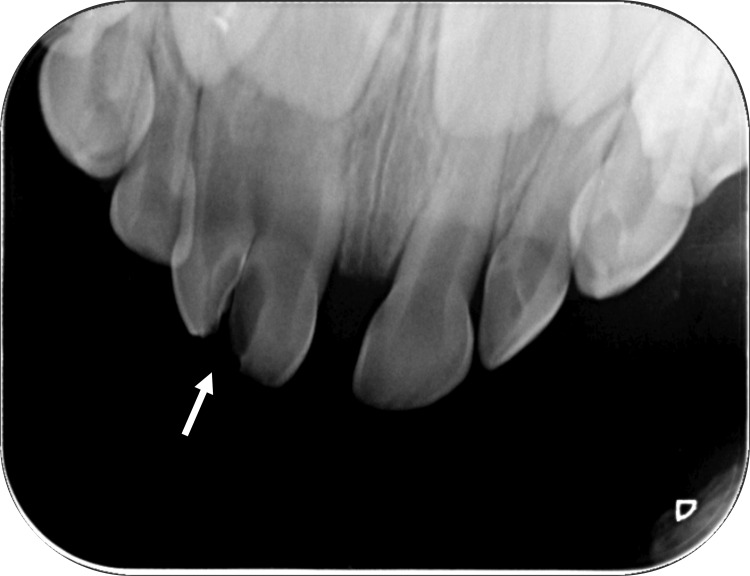
Occlusal radiograph (maxillary anterior region, occlusal view). Two separate root canals and two completely fused roots are visible within the anomalous tooth (arrow). The permanent successor (tooth 11) is identified in its expected position with no evidence of displacement or follicular enlargement.

Diagnosis

The diagnosis of fusion of the primary maxillary right central incisor (51) with a mesiodens was established based on the clinical and radiographic evidence of a single tooth with a wider crown mesiodistally, a deep groove, two separate root canals, and two roots. Although the number of teeth, counting the anomalous tooth as one, remained normal, considering the morphology of the crown and the root, gemination was ruled out, as the findings indicated a tooth fused with a supernumerary tooth rather than the division of a single tooth germ [8].

Treatment

The treatment options were discussed with the parents, including conservative management with endodontic treatment, surgical separation, and extraction. Considering the age of the patient, the natural exfoliation of the tooth, the extent of the lesions, and the expected natural eruption of the successor, extraction was chosen. The fused tooth was extracted atraumatically under local anesthesia (infiltration with 4% articaine with 1:100,000 epinephrine) using standard pediatric extraction forceps. The extraction was performed without complications, and the surgical site was inspected to ensure complete removal of the fused roots (Figure 3). Postoperative instructions were provided, including a soft diet, oral hygiene, and analgesics prescribed as needed (PRN) for postoperative discomfort.

**Figure 3:**
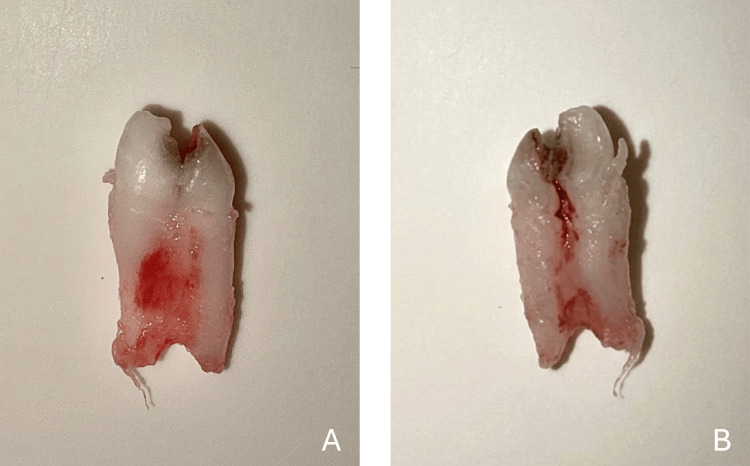
Photograph of the extracted fused tooth (primary right central incisor 51 fused with mesiodens) following removal. A) Labial view. (B) Palatal view. The single crown unit with dual root morphology is consistent with the radiographic findings of two separate root canals and two fused roots, confirming the diagnosis of dental fusion rather than gemination.

Follow-up

A long-term follow-up plan was established. The patient was examined at one week post-extraction, confirming satisfactory soft-tissue healing. Subsequent clinical and radiographic reviews were scheduled at 6, 12, and 24 months to monitor eruption of the permanent successor and detect any signs of agenesis or ectopic eruption.

## Discussion

This report presents a case of fusion between a primary maxillary central incisor and a mesiodens in a 3.5-year-old boy. While the prevalence of fusion in the primary dentition and mesiodens is documented individually, to our knowledge, no epidemiological study has reported the prevalence of fusion between a primary tooth and a mesiodens.

This case is also noteworthy for the following reasons. First, the majority of published cases of fusion with a mesiodens involve permanent teeth [1,9], while our case involves primary dentition. Furthermore, fused teeth are often detected incidentally; therefore, symptoms such as pain or sensitivity suggest carious lesions alongside the groove of the fusion or pulpal involvement [2,7]. Lastly, the young age of the patient (3.5-year-old) highlights the importance of clinical examination even in early primary dentition.

The differential diagnosis between fusion and gemination remains challenging when supernumerary teeth are involved [8]. The tooth-counting method is useful, as counting the anomalous tooth as one, in gemination the number is normal, while in fusion it appears as one missing. In the case where a supernumerary tooth is involved, the count of teeth in fusion remains normal as well. Advanced radiographic imaging, such as CBCT, provides valuable information about the anatomy of pulp and root canals, facilitating both diagnosis and treatment planning [9,10]. The use of CBCT in primary dentition should be weighed against radiation exposure; therefore, in our case, the conventional occlusal radiograph was selected, providing sufficient information for our diagnosis. Recent studies show CBCT provides superior anatomic detail for anterior tooth anomalies compared with conventional radiographs, although its use in young children must be balanced against radiation exposure (ALARA Principle) [11,12].

Fused primary teeth management depends on clinical evidence. A systematic review by Bernardi et al. found that treatment of fusion can be preventive or surgical based on caries and malocclusion risk [13]. Santos et al. in 2003 published a comparable case of fusion between a primary central incisor and a supernumerary tooth in a four-year-old girl that was managed conservatively with endodontic and restorative treatment [14]. On the contrary, our case required extraction due to pain symptoms and extent of the pathology. Conservative management with endodontic treatment would have required pulpectomy of both root canals given the symptomatic pulpal involvement, followed by coronal restoration. This demanding procedure in a 3.5-year-old with limited cooperation could have low success rates, given the imminent natural exfoliation of the tooth. Surgical separation of the fused components before individual treatment has also been described in the literature but has a risk of injury to the underlying permanent tooth germ, particularly in young patients where the successor is in close proximity. Given the combination of symptomatic pulpal involvement, extensive carious lesions, young patient age, and proximity of the permanent successor, extraction represented the most predictable management option with the lowest risk of harm to the developing dentition. A critical aspect after the extraction is the effect on the permanent successor. Fusion of primary teeth has been associated with agenesis of the permanent tooth in 50%-61% of the cases [4,15]. Regular clinical and radiographic examination is important to evaluate the eruption of the permanent successor for better management planning in case of any abnormality.

Acharya et al. reported fusion of a primary central incisor with a supernumerary tooth in a seven-year-old boy [3], and Goswami and Lohia in 2024 reported fusion of primary teeth in two pediatric patients, though in their cases the fusion was between adjacent teeth and not between a tooth and a mesiodens [16]. Compared with the existing literature, our case is distinctive in combining the youngest reported patient age, specific involvement of a mesiodens rather than an adjacent tooth, and a symptomatic presentation that required extraction rather than conservative management.

## Conclusions

Fusion of a primary maxillary central incisor with a mesiodens is a rare developmental anomaly that can occur in early primary dentition. Detailed clinical examination alongside radiographic imaging is essential for precise diagnosis and differentiation from gemination. Extraction is a viable management choice in cases with symptoms of pain in primary dentition. Long-term follow-ups (clinical and radiographic review at 6, 12, and 24 months) are crucial for evaluating the eruption of the permanent teeth and early detection of other consequences such as agenesis of the successor tooth or orthodontic problems.
